# Transmembrane routes of cationic liposome-mediated gene delivery using human throat epidermis cancer cells

**DOI:** 10.1007/s10529-013-1325-0

**Published:** 2013-09-26

**Authors:** Shaohui Cui, Bing Wang, Yinan Zhao, Huiying Chen, Huiqin Ding, Defu Zhi, Shubiao Zhang

**Affiliations:** SEAC-ME Key Laboratory of Biotechnology and Bio-resouces Utilization, College of Life Science, Dalian Nationalities University, Dalian, 116600 Liaoning China

**Keywords:** Cancer cells, Cationic liposome, Endocytic pathway, Gene delivery, Human throat cancer cells, Inhibitor, Transfection efficiency

## Abstract

For studying the mechanism of cationic liposome-mediated transmembrane routes for gene delivery, various inhibitors of endocytosis were used to treat human throat epidermis cancer cells, Hep-2, before transfection with Lipofectamine 2000/pGFP-N2 or Lipofectamine 2000/pGL3. To eliminate the effect of inhibitor toxicity on transfection, the RLU/survival rate was used to represent the transfection efficiency. Chlorpromazine and wortmannin, clathrin inhibitors, decreased transfection efficiency by 44 % (100 μM) and 31 % (100 nM), respectively. At the same time, genistein, a caveolin inhibitor, decreased it by 30 % (200 μM). Thus combined transmembrane routes through the clathrin and caveolae-mediated pathways were major mechanisms of cell uptake for the cationic liposome-mediated gene delivery. After entering the cells, microtubules played an important role on gene delivery as vinblastine, a microtubulin inhibitor, could reduce transfection efficiency by 41 % (200 nM).

## Introduction

Although non-viral vectors are inferior compared to viral vectors in terms of transfection efficiency, they have the advantages over viral ones as they are non-immunogenic, easy to produce and are not oncogenic. Among non-viral vectors, cationic liposomes are used extensively as gene carriers for plasmid DNA (pDNA), antisense oligonucleotides and small interfering RNA (High 2005; Obata et al. 2010). Because the cell membrane is the first barrier for liposome/DNA complexes (lipoplexes) to enter into cells, the mechanism of cationic liposome-mediated transmembrane gene delivery is important as a study of it could contribute to improving transfection efficiency.

Endocytosis is generally considered to be the main entering pathway for lipoplexes (Chittimalla et al. 2005; Payne et al. 2007). There are however, various endocytic pathways in eukaryotic cells, such as clathrin-dependent and clathrin-independent pathways. The latter includes phagocytosis, macropinocytosis and caveolae-mediated internalization (Sandvig et al. 2011). The relative contribution of each pathway in lipoplexes internalization has been poorly defined to date (Mayor and Pagano 2007; Howes et al. 2010). Endocytotic inhibitors, such as chloroquine, chlorpromazine, NaN_3_ or filipin, are helpful for studying drug intracellular release or elucidating a specific endocytosis route (Blanchard et al. 2006). There are still many problems that have not been elucidated on the biological barriers although much effort has been expended on them. As non-viral vectors are less efficient than the viral ones, therefore understanding the mechanism of transmembrane might contribute to overcoming the hurdles in gene delivery. This study was aimed to elucidate the transmembrane pathways of gene delivery mediated by a cationic liposome, Lipofectamine 2000, while the inhibitors such as chlorpromazine (wortmannin), genistein and vinblastine for inhibiting clathrin, caveolin and microtubulin pathways, respectively, were used to treat Hep-2 cells before transfection.

## Materials and methods

### Materials and reagents

Human throat epidermis (Hep-2) cancer cell line was purchased from Cell Bank of Chinese Academy of Sciences. Lipofectamine 2000 was from Invitrogen. The pGL3-control (pLuc) vector plasmid (Promega) containing a modified coding region for firefly (*Photinus pyralis*) luciferase and the pGFP-N2 vector plasmid (Clontech) containing a coding region for green fluorescence protein were used for transfection. Bright-Glo Luciferase Assay System was purchased from Promega.

### Cell culture

Hep-2 cells were grown in a 100 ml culture flask in RPMI-1640 medium supplemented with 10 % (v/v) FBS and antibiotics (penicillin 100 U/ml and streptomycin 100 U/ml) at 37 °C in a humidified atmosphere containing 5 % (v/v) CO_2_.

### Plasmid preparation

pGL3-control and pGFP-N2 plasmids were amplified in *Escherichia coli* (strain JM109 and DH5-a, respectively) and purified using an endotoxin-free kit (Qiagen) to remove the bacterial endotoxins. The quantity and quality of purified pDNA were assessed at 260 and 280 nm by electrophoresis in agarose gels.

### Lipoplexes preparation

For the preparation of lipoplexes, 0.3 μl pDNA (pGFP-N2 or pGL3) (1 mg/ml) and 0.6 μl Lipofectamine 2000 (1 mg/ml), were diluted in Opti-MEM medium 25 μl, respectively, and then the diluted transfection reagent was added to pDNA solutions. The mixtures were vortexed gently and incubated for 20 min at room temperature for 96 well plate transfection.

### Transfection procedure

Hep-2 cells (10^5^ cells/ml) were seeded into 96-well plates in 100 μl growth medium (RPMI-1640) until the required cell number was obtained (80 % confluence) at the time of transfection. Cells were washed once with PBS, and then 50 μl lipoplexes (prepared as described above) were added to each well. It was mixed gently by rocking the plate. The cells were incubated for 4–6 h, washed by PBS once and the growth medium was replaced. Cells were further cultured for 48 h prior to analysis.

### Luciferase assay

Relative luciferase activity was assessed using the Bright-Glo Luciferase Assay System and a microplate reader. The growth medium was removed from each well, cells were rinsed once with PBS and luciferase activity was measured after 100 μl lysis buffer was added into each well of the 96-well plate with the incubation of 5 min at room temperature. The protein concentrations of cell lysates per well were determined using the BCA protein assay kit, and then the transfection efficiency was obtained as the relative luciferase activity. Data were expressed as relative light units (RLU) per mg protein. Each data point was averaged over three replicates.

### Green fluorescence protein (GFP) assay

The expression of GFP was imaged by inverted fluorescence microscope and transfection efficiency was given as relative efficiency. The number of GFP-expressing cells versus the total cell quantity in the microscope was defined as the transfection efficiency. Cell counting was performed randomly in microscopic observation scope under 10 × 20 magnification with three repeats.

### Cytotoxicity assay

The cytotoxicity was evaluated by MTT assay. Hep-2 cells (10^5^ cells/ml) were seeded into 96-well plates at 200 μl per well and incubated 24 h before treatment. Various kinds of inhibitors were added to the cells and after 1 h, 50 μl MTT (5 mg/ml in PBS) was added and incubated for an additional 4 h. MTT-containing medium was removed and 150 μl DMSO was added to dissolve the formazan and the absorbance was measured at 570 nm to determine cell survival as percentage of control. Data were presented as the mean ± SD.

## Results

### The effects of clathrin inhibitors on transfection of lipoplexes

**Fig. 1:**
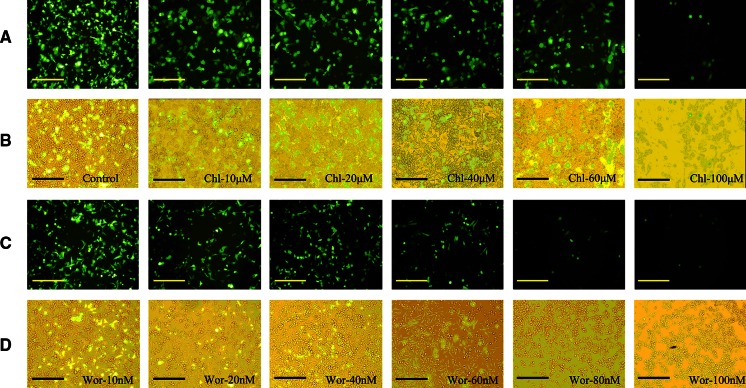
Effect of concentrations of chlorpromazine (10–100 μM) and wortmannin (10–100 nM) on GFP expression mediated by Lipofecamine 2000/pGFP-N2 complexes against Hep-2 cells. **a**, **c** The expression of GFP was imaged by inverted fluorescence microscope (10 × 20); **b**, **d** contrast cells in bright field. *Scale bar* = 50 μm

Endocytosis is the main process by which cells take up macromolecules. However, there are many types of endocytic pathways, among which the so-called clathrin-dependent endocytosis is well characterized. To determine the influence of clathrin-dependent endocytosis on cationic liposome-mediated transfection, two inhibitors (chlorpromazine and wortmannin) of clathrin were used to treat Hep-2 cells before transfection. The transfection efficiency of Lipofectamine 2000/pGFP-N2 complexes was investigated through the detection of GFP expression (Fig. 1). GFP expression obviously decreased with increasing inhibitor concentrations. When chlorpromazine was 100 μM, GFP expression was very low (Fig. 1a, b). In the same way, there was significant inhibition when wortmannin was higher than 60 nM (Fig. 1c, d).

**Fig. 2:**
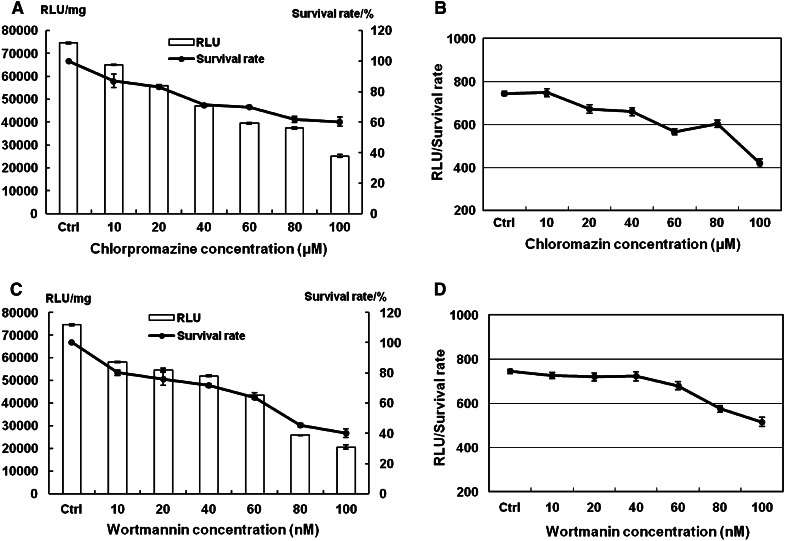
Luciferase reporter gene expression and cell survival rate. **a**, **c** The evaluation of transfection efficiency and cell survival rate with chlorpromazine (10–100 μM) and wortmannin (10–100 nM) treated Hep-2 cells. Lipofectamine 2000 was used as positive control in transfection. Cell survival rate was determined with the MTT method with non-treated cells as control. All results are expressed as mean ± SD (*n* = 4). **b**, **d** RLU/survival rate with chlorpromazine (10–100 μM) and wortmannin (10–100 nM) treated Hep-2 cells

Additionally, luciferase reporter gene was used to examine the effects of inhibitors on transfection efficiency. Luciferase activity at 48 h after transfection was measured (Fig. 2): luciferase gene expression, as RLU in Hep-2 cells, was reduced by up to 66 % when cells were treated with up to 100 μM chlorpromazine (Fig. 2a) and by up 72 % when cells were treated with up to 100 nM wortmannin (Fig. 2c).

The cytotoxicity of chlorpromazine and wortmannin was tested by MTT assay, as these inhibitors could influence cell survival and evaluation of the transfection efficiency. Cell survival rates were between 87 and 60 % when cells were treated cells chlorpromazine (Fig. 2a), and between 79 and 41 % with wortmannin (Fig. 2c). To minimize the influence of toxicity of the inhibitors on transfection, the ratios of RLU to survival rate were used to show the inhibition of transfection. The RLU/survival rate also decreased with an increase of chlorpromazine and wortmannin concentrations. The expression of protein was reduced by up to 44 % of the RLU/survival rate with chlorpromazine up to 100 μM (Fig. 2b). For wortmannin, expression was reduced by up to 31 % at 100 nM (Fig. 2d). These results suggested that both chlorpromazine and wortmannin lower transfection efficiencies by inhibiting clathrin**-**dependent endocytosis.

### The effects of caveolin inhibitor on transfection of lipoplexes

**Fig. 3:**
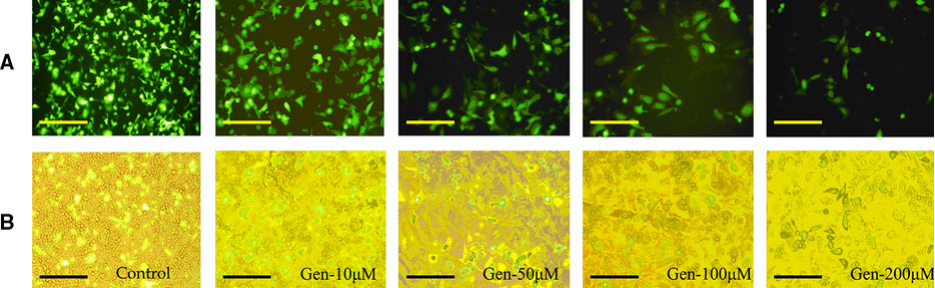
Effect of concentrations of genistein (10–200 μM) on GFP expression mediated by Lipofecamine 2000/pGFP-N2 complexes. **a** Inverted fluorescent microscope images (10 × 20); **b** contrast cells in bright field. *Scale bar* = 50 μm

**Fig. 4:**
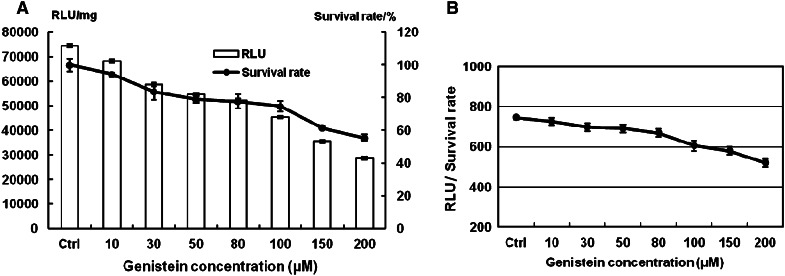
Luciferase reporter gene expression and cell survival rate. **a** The evaluation of transfection efficiency and cell survival with genistein (10–200 μM) treated Hep-2 cells. Lipofectamine 2000 was used as positive control. Cell survival rate was determined with the MTT method with non-treated cells as control. All results are expressed as mean ± SD (*n* = 4). **b** RLU/survival rate with genistein (10–200 μM) treated Hep-2 cells

Besides clathrin-dependent endocytosis, another internalization pathway is caveolae-dependent. Genistein, the inhibitor of caveolin, which is essential for the formation and stability of caveolae, was used to treat Hep-2 cells 1 h before transfection. Fig. 3 shows that the transfection efficiency, in terms of GFP expression, was reduced by up to 80 % with up to 200 μM genistein. Luciferase reporter gene expression showed that transfection efficiency was reduced by up to 62 % with genistein up to 200 μM compared to the control (Fig. 4a). The cytotoxicity of genistein to cells is shown in Fig. 4a. The ratios of RLU to survival rate showed the protein expression was decreased by 30 % with 200 μM genistein (Fig. 4b). These results demonstrated the route of transmembrane transport of lipoplexes by caveolae-dependent pathway also played an important role.

### The effects of microtubule inhibitor on transfection of lipoplexes

**Fig. 5:**
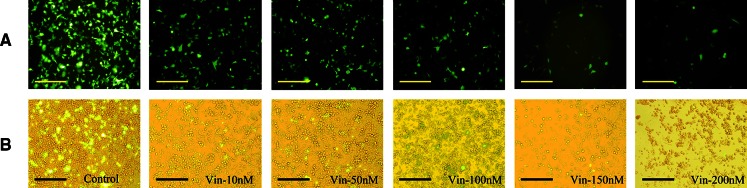
Effect of concentrations of vinblastine (10–200 nM) on GFP expression mediated by Lipofecamine 2000/pGFP-N2 complexes. **a** Inverted fluorescent microscope images (10 × 20); **b** contrast cells in bright field. *Scale bar* = 50 μm

**Fig. 6:**
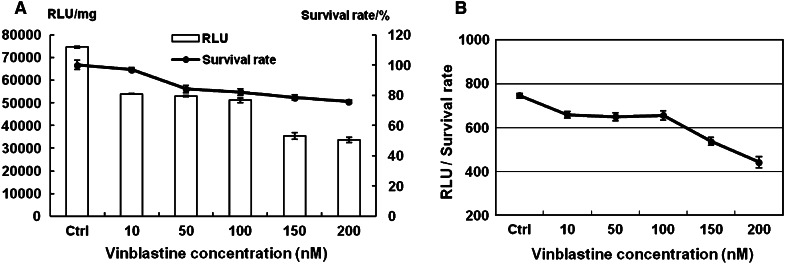
Luciferase reporter gene expression and cell survival rate. **a** The evaluation of transfection efficiency and cell viability with vinblastine (10–200 nM) treated Hep-2 cells. Lipofectamine 2000 was used as positive control. Cell viability was determined with the MTT method with non-treated cells as control. All results are expressed as mean ± SD (n = 4). **b** RLU/survival rate with vinblastine (10–200 nM) treated Hep-2 cells

Uptake of materials into cells and subsequent internalization is usually related to the function of microtubules. Some proteins are trafficked to the nucleus by microtubules and are further dismantled in cells. Vinblastine, which is an inhibitor of microtubule function, was used to treat cells 1 h before transfection. Then, in the same way, transfected cells were detected after the delivery of the reporter genes. The transfection efficiency of pGFP-N2 was reduced by up to 98.3 % when cells were treated with up to 200 nM vinblastine (Fig. 5). The results of luciferase reporter gene expression showed that transfection efficiency was reduced by up to 54 % with vinblastine up to 200 nM (Fig. 6a). The cytotoxicity of vinblastine to cells is shown in Fig. 6a. RLU/survival rate (Fig. 6b) showed the expression of luciferase gene was reduced by up to 41 % with vinblastine up to 200 nM. These results indicated that vinblastine could reduce the transfection efficiency of lipoplexes through inhibiting the function of microtubulin.

## Discussion

Knowledge about the transmembrane uptake mechanism is important for the development of efficient carriers for gene delivery. So far, some results indicate that endocytosis is the major mechanism of lipoplexes entry into cells. There are various endocytic pathways in eukaryotic cells, among them clathrin-mediated endocytosis functions by means of coated pits and endocytic internalization. Clathrin-independent endocytic pathways include phagocytosis, macropinocytosis and caveolae-mediated endocytosis (Payne et al. 2007; Ziello et al. 2010). But there are few studies to focus on the detailed transporting routes of cationic liposome-mediated gene delivery. This study aimed to elucidate this problem by using different inhibitors to inhibit the pathways of cell uptake, hoping to provide reference for better understanding and overcoming the biological barriers which exist in the process of gene delivery.

von Gersdorff et al. (2006) showed that chlorpromazine inhibition of the clathrin-dependent uptake route resulted in the loss of transfection with PEI polyplexes in several cell lines, suggesting that the clathrin-dependent uptake route is important for successful transfection. However, combined pathways may function at the same time. So we used several inhibitors to study cationic liposome-mediated cell uptake pathways. The results showed that chlorpromazine and wortmannin could reduce transfection efficiency by 30–40 % in terms of RLU/survival rate by inhibiting clathrin route. Chlorpromazine inhibits uptake pathway mediated via inhibiting clathrin-coated nest aggregation in the membrane. Wortmannin inhibits uptake by affecting the formation of double-shell of the endosomes to reduce the intracellular endosomal vesicles (van der Aa et al. 2007). Genistein, as inhibitor of caveolin, reduced transfection efficiency by ~30 %. Vinblastine could reduce 40 % of transfection efficiency of lipoplexes by inhibiting the function of microtubulin.

Our results indicated that transfection efficiency was decreased after incubation with inhibitors, both through the clathrin- and caveolae-mediated pathways and also through microtubulin. A correlation between clathrin-mediated or caveolae-mediated endocytosis and lipoplexes transfection was supported convincingly by several lines of evidence, including the use of inhibitors of endocytosis, co-localization with pathway-specific markers, and deficiencies in clathrin-mediated endocytosis. Additionally, the inhibition of microtubulin probably changed the microtubule function for lipoplexes transport through microtubule polymerization by the addition of vinblastine. Cell uptake and subsequent internalization is usually related to the function of microtubule. The cytoskeleton is required for the formation and internalization of clathrin-coated pits (Yarar et al. 2005). Vinblastine can affect the actin cytoskeleton grouping into actin filament polymerization orderly and promote its dissociation. Therefore, it can inhibit the uptake pathway mediated by microtubule and microfilament.

The use of inhibitors might give rise to an unambiguous outcome because of toxic side-effects (Chittimalla et al. 2005). So we took the cell survival rate into account during the uptake study of lipoplexes into cells. The ratios of RLU to survival used in this paper, could better address the problem caused by the toxicity of inhibitors.

Taken together, these results demonstrated that the uptake of lipoplexes to Hep-2 cells was mediated via both the clathrin-mediated and caveolae-mediated endocytic pathways. Otherwise, phagocytosis mediated by microtubule-microfilament also played a role in the regulation. There is no doubt that the combined action of these endocytic pathways is essential for gene delivery, and their roles will be clarified further as the studies continue. Though we need to further elucidate the functions of inhibitors and more detailed mechanism of liposomes mediated transfection, the results of this article could provide reference for the acquisition of novel gene delivery systems.
